# Uveitis-Glaucoma-Hyphema Syndrome Secondary to Asymmetric Intraocular Lens (IOL) Haptic Fixation: A Clinical Lesson in Mechanism-Based Diagnosis and Treatment

**DOI:** 10.7759/cureus.110320

**Published:** 2026-06-05

**Authors:** Tommaso Bonifazi, Masaki Tanito

**Affiliations:** 1 Department of Ophthalmology, Shimane University Faculty of Medicine, Izumo, JPN

**Keywords:** ahmed glaucoma valve, anterior segment optical coherence tomography, intraocular lens, laser flare meter, small-incision cataract surgery, uveitis-glaucoma-hyphema (ugh) syndrome

## Abstract

Uveitis-glaucoma-hyphema (UGH) syndrome is a rare complication of cataract surgery caused by mechanical chafing between the intraocular lens (IOL) and adjacent uveal structures. We report a case of UGH syndrome caused by inadvertent sulcus placement of an IOL haptic. An 80-year-old man developed progressive ocular hypertension after apparently uncomplicated cataract surgery and was treated with topical antiglaucoma therapy. Approximately 30 months after cataract surgery, hyphema and decreased vision occurred, and the patient was referred to our institution. Slit-lamp examination showed mild nasal IOL decentration, while gonioscopy revealed marked inferior angle pigmentation. Anterior segment optical coherence tomography (AS-OCT) demonstrated IOL tilt and contact between the posterior iris surface and the nasal haptic. The patient underwent repositioning of the displaced haptic into the capsular bag, combined with Ahmed glaucoma valve implantation and pars plana tube insertion. After surgery, AS-OCT confirmed resolution of iris-IOL contact, and intraocular pressure was well controlled. This case may serve as a lesson for residents and young ophthalmologists who may encounter ocular hypertension of unclear origin, emphasizing the need to maintain a high index of suspicion for UGH syndrome. Careful anterior segment examination, together with AS-OCT, may help identify occult iris-IOL chafing and guide appropriate surgical management.

## Introduction

Originally described by Ellingson in 1978 in association with anterior chamber intraocular lenses (ACIOLs) [1], uveitis-glaucoma-hyphema (UGH) syndrome is an uncommon complication of cataract surgery characterized by intraocular inflammation, hyphema, and elevated intraocular pressure (IOP) [2]. The underlying mechanism is thought to involve mechanical chafing between the intraocular lens (IOL) and adjacent uveal structures, leading to recurrent bleeding, pigment dispersion, inflammation, and secondary trabecular meshwork dysfunction [2]. Since the introduction of posterior chamber intraocular lenses (PCIOLs) and advances in IOL design and surgical technique, the incidence of UGH syndrome has markedly decreased, with reported rates ranging from 0.4% to 1.2% [2,3]. However, cases associated with PCIOLs continue to be reported, particularly in eyes with subtle IOL malposition or instability [4-7]. Because symptoms can arise months or years after apparently uncomplicated surgery, diagnosis is often challenging and may be delayed until glaucomatous damage has already developed [2,3].

We report a case of UGH syndrome in a patient who developed progressive ocular hypertension following apparently uncomplicated cataract surgery. The patient was initially treated with topical antiglaucoma therapy, but the underlying mechanism remained unclear until careful anterior segment examination and anterior segment optical coherence tomography (AS-OCT) revealed asymmetric haptic fixation with iris-IOL chafing caused by a haptic displaced into the ciliary sulcus.

## Case presentation

An 80-year-old man had undergone apparently uneventful small-incision cataract surgery with implantation of a single-piece IOL (SN60AT +21.5 D, Alcon Japan, Tokyo, Japan) in his right eye (OD) by a local ophthalmologist. Six months after surgery, best-corrected visual acuity (BCVA) was 1.2, and IOP was 17 mmHg. During the following months, the patient developed progressive ocular hypertension and was treated by the same physician with escalating topical antiglaucoma therapy, requiring up to three medications. Approximately 30 months after cataract surgery, the patient developed sudden visual loss associated with hyphema and was referred to our institution for further evaluation.

**Figure 1:**
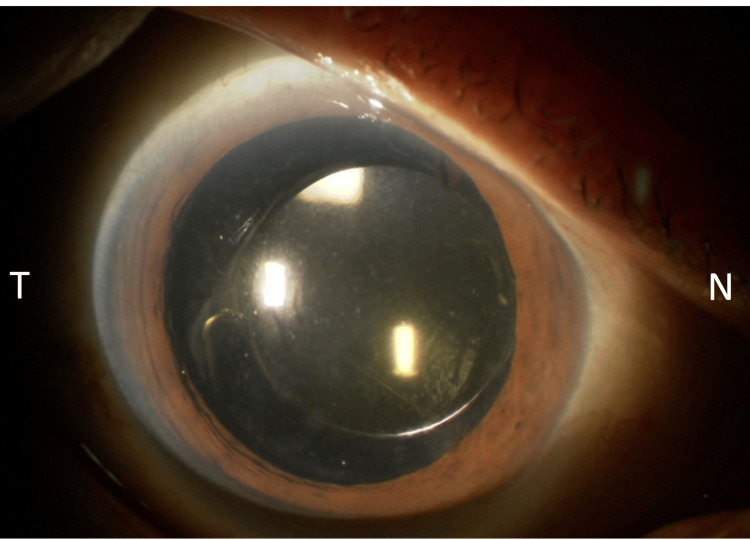
Preoperative slit-lamp photograph of the right eye Slit-lamp photograph showing mild nasal decentration of the IOL. IOL, intraocular lens

**Figure 2:**
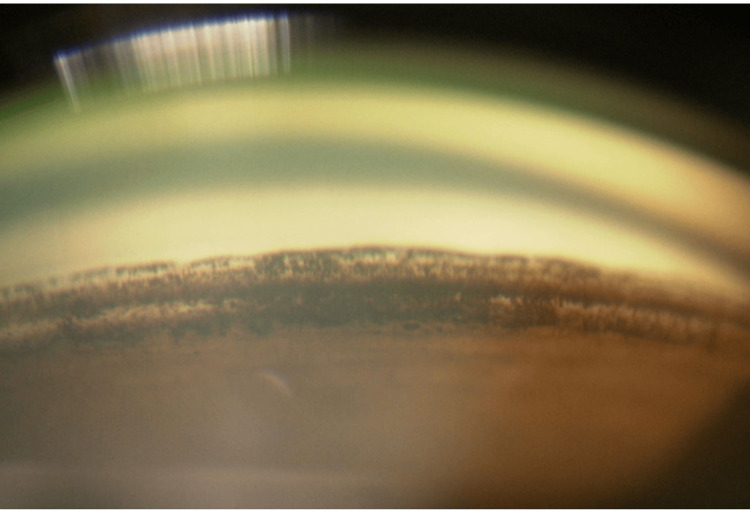
Preoperative gonioscopy of the right eye Gonioscopy demonstrating marked inferior angle pigmentation.

**Figure 3:**
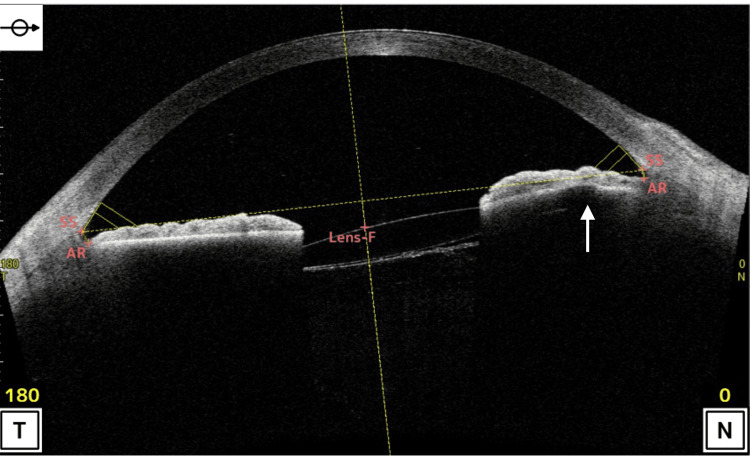
Preoperative AS-OCT of the right eye Horizontal scan demonstrating IOL tilt and distortion of the nasal iris profile caused by contact between the nasal haptic and the posterior iris surface (arrow). AR, angle recess; AS-OCT, anterior segment optical coherence tomography; IOL, intraocular lens; Lens-F, anterior lens surface; N, nasal; SS, scleral spur; T, temporal

At presentation, BCVA was 0.4, and IOP was 32 mmHg. Slit-lamp examination revealed mild nasal decentration of the IOL (Figure 1), while gonioscopy showed marked inferior angle pigmentation (Figure 2). At the time of presentation to our hospital, the hyphema had already resolved. AS-OCT (Casia 2, Tomey Corporation, Nagoya, Japan) demonstrated IOL tilt and distortion of the nasal iris profile caused by contact between the nasal haptic and the posterior iris surface (Figure 3, arrow). Anterior chamber flare measured with a laser flare meter (FM-600, Kowa, Tokyo, Japan) was 45.0 photon counts (pc)/msec OD, compared with 9.1 pc/msec in the fellow eye. Corneal endothelial cell density measured by specular microscopy (EM-3000, Tomey Corporation) was 1426 cells/mm² OD and 2342 cells/mm² in the fellow eye. Humphrey 30-2 visual field testing (Humphrey Field Analyzer, Carl Zeiss Meditec, Dublin, CA, USA) showed a mean deviation of -6.75 dB.

**Figure 4:**
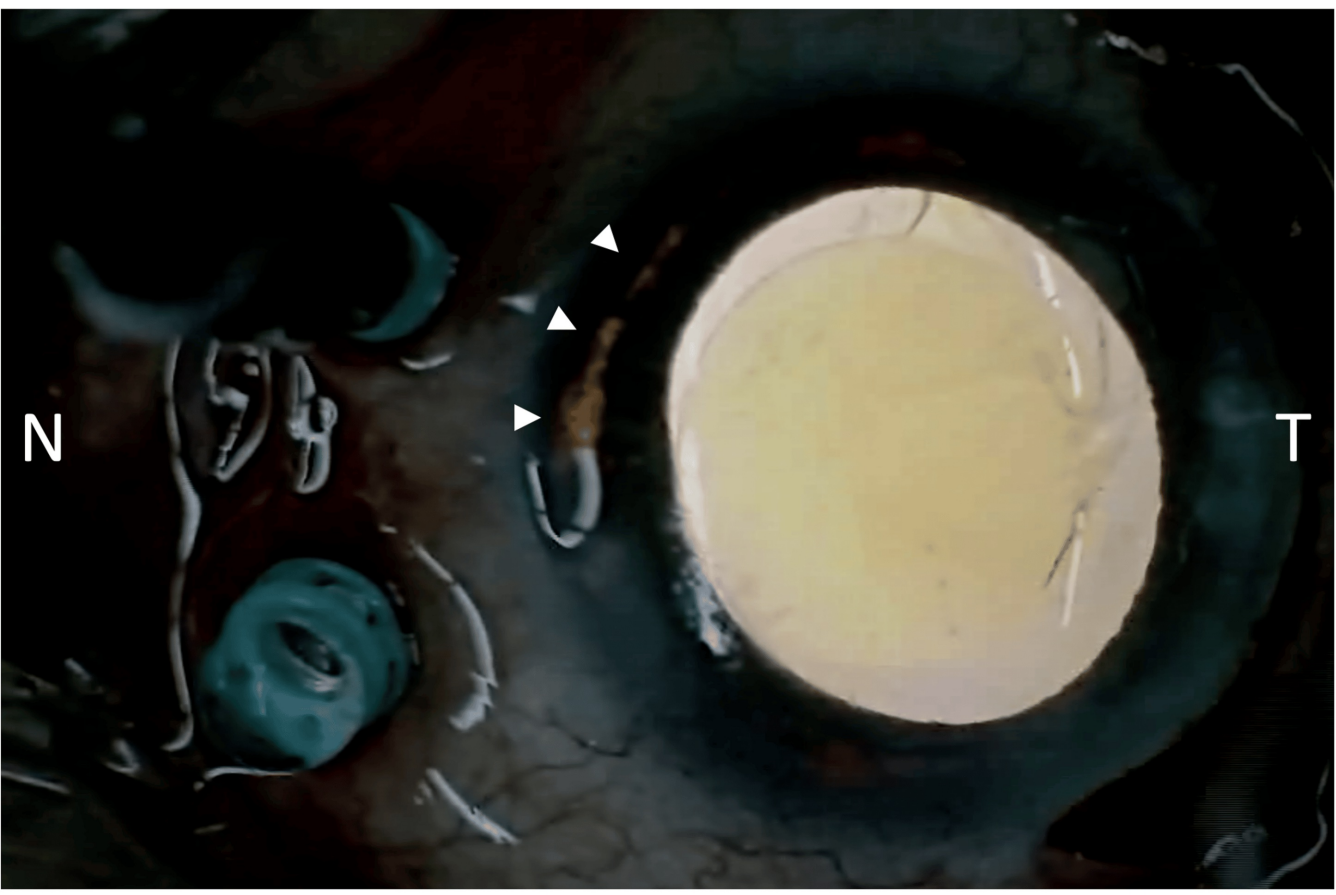
Intraoperative transillumination photograph of the right eye Intraoperative photograph showing a transillumination defect in the nasal iris at the site of iris-haptic contact (arrowheads).

A diagnosis of UGH syndrome secondary to asymmetric IOL haptic fixation was made, and the patient underwent IOL repositioning combined with AGV implantation (model AGV FP-7, JFC Sale Plan Co., Ltd., Tokyo, Japan). Intraoperatively, the nasal haptic was found to be displaced into the ciliary sulcus, whereas the temporal haptic was within the capsular bag. An iris transillumination defect was observed at the site of iris-haptic contact (Figure 4). The nasal haptic was repositioned into the capsular bag, and the IOL was recentered. An AGV was implanted in the superotemporal quadrant, and 25-gauge pars plana vitrectomy was performed for pars plana tube insertion (Video 1). The surgical technique for AGV implantation has been described previously [8]. The postoperative course was uneventful.

**Video 1:** Surgical management of UGH syndrome secondary to asymmetric haptic fixation Surgical video demonstrating repositioning of the displaced nasal haptic into the capsular bag, combined with Ahmed glaucoma valve implantation and 25-gauge pars plana vitrectomy for pars plana tube insertion. UGH, uveitis-glaucoma-hyphema

**Figure 5:**
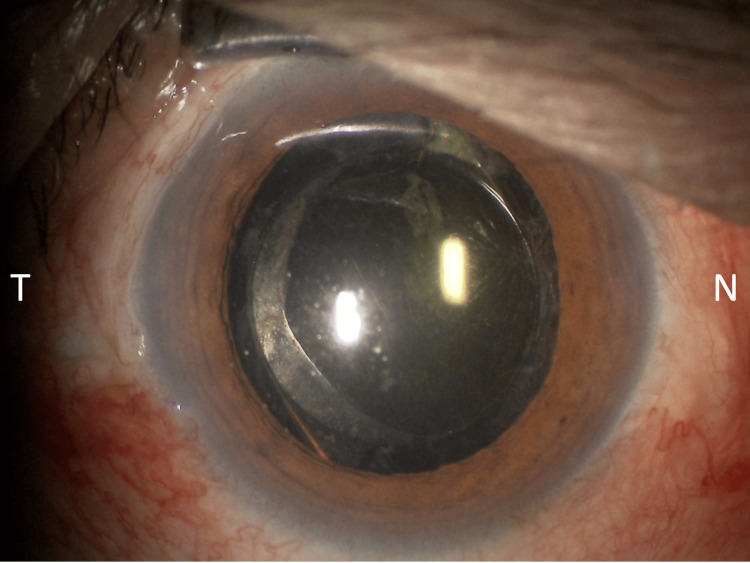
Postoperative slit-lamp photograph of the right eye Postoperative slit-lamp photograph showing a well-centered IOL after repositioning of the displaced nasal haptic into the capsular bag. IOL, intraocular lens

**Figure 6:**
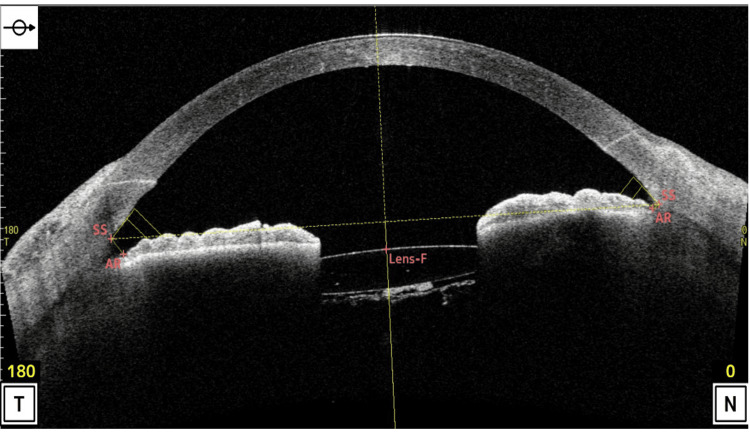
Postoperative AS-OCT of the right eye Horizontal scan confirming resolution of the iris-IOL contact after repositioning of the nasal haptic. AR, angle recess; AS-OCT, anterior segment optical coherence tomography; IOL, intraocular lens; Lens-F, anterior lens surface; N, nasal; SS, scleral spur; T, temporal

At the three-month follow-up visit, BCVA was 0.4, and IOP was 13 mmHg on one antiglaucoma medication. The IOL appeared well centered (Figure 5), and AS-OCT showed resolution of the iris-IOL contact (Figure 6). Anterior chamber flare was 24.9 pc/msec OD.

## Discussion

Although originally described in association with ACIOLs, cases of UGH syndrome continue to be reported after the introduction of modern PCIOLs [4-7]. Diagnosis may be challenging, particularly when symptoms occur without a clear temporal relationship to prior cataract surgery, potentially mimicking hypertensive uveitis, postoperative inflammation, or other forms of secondary glaucoma [4]. In the present case, the patient developed progressive ocular hypertension after apparently uncomplicated cataract surgery and was treated with escalating topical antiglaucoma therapy. The underlying mechanism remained unrecognized for months until sudden visual loss associated with hyphema prompted referral to our institution. Slit-lamp examination raised suspicion of iris-IOL chafing, while AS-OCT demonstrated IOL tilt and contact between the posterior iris surface and the nasal haptic, which appeared to be displaced into the ciliary sulcus. Previous studies have highlighted the role of AS-OCT in diagnostically challenging cases of UGH syndrome [9,10]. Nevertheless, because the high reflectivity of the iris pigment epithelium limits visualization of structures posterior to the iris, AS-OCT findings should always be interpreted in the context of the clinical history and slit-lamp examination.

Proposed mechanisms of UGH syndrome associated with PCIOLs include IOL instability and late spontaneous IOL dislocation, which have been reported more frequently in eyes with pseudoexfoliation syndrome, trauma, or other conditions associated with zonular weakness [5-7,11]. In the present case, however, there was no evidence of zonular instability. We therefore suspect that the nasal haptic had been inadvertently positioned in the ciliary sulcus at the time of cataract surgery, causing chronic iris-IOL chafing, subclinical inflammation, and pigment dispersion. Interestingly, in a postmortem study, Apple et al. identified asymmetric haptic fixation in nearly half of pseudophakic eyes with apparently in-the-bag IOL implantation [12], suggesting that this configuration may be more common than generally recognized. However, the study was conducted before the widespread adoption of modern phacoemulsification techniques and included eyes implanted with older-generation PCIOLs; therefore, its findings may not reflect the prevalence observed with modern PCIOLs and contemporary phacoemulsification techniques. The development of symptoms after sulcus fixation may also depend on IOL design [1,2]. In the present case, the implanted PCIOL had a sharp posterior optic edge, which may have contributed to chronic iris irritation.

Elimination of the mechanical source of iris chafing remains essential in the management of UGH syndrome [13]. In our patient, the capsular bag appeared intact and stable, allowing repositioning of the displaced haptic into the capsular bag. However, this approach may not be feasible in eyes with marked capsular fibrosis, extensive adhesions, poor capsular support, or previous posterior Nd capsulotomy, and in some cases, IOL explantation and exchange may be preferable. Because of the severity of ocular hypertension, the presence of glaucomatous visual field damage, and the marked trabecular pigmentation, IOL repositioning was combined with AGV implantation. At the three-month follow-up visit, the IOL appeared well centered, and IOP was controlled.

## Conclusions

This case highlights the diagnostic challenges of UGH syndrome caused by asymmetric IOL haptic fixation and shows how it may be easily overlooked, leading to progressive ocular hypertension and irreversible glaucomatous damage. Careful anterior segment examination, together with AS-OCT, may help identify occult iris-IOL chafing and guide appropriate surgical management. We believe that this case may serve as a lesson for residents and young ophthalmologists managing pseudophakic patients with unexplained ocular hypertension, reminding them to maintain a high index of suspicion for UGH syndrome even years after apparently uncomplicated cataract surgery.
